# Supplementary material for: Genetic Diversity of Enteric Viruses in Children under Five Years Old in Gabon
Source: Viruses. 2021 Mar 24;13(4):545. doi: 10.3390/v13040545 (PMC8064335; doi:10.3390/v13040545)
Supplement: Supplementary file 1 [file viruses-13-00545-s001.zip › viruses-111060_Supplementary table 2_revised.docx]

Supplementary table 2. Hitherto unpublished PCR programs and primers used in this study

| assay | PCR program | primer |
| --- | --- | --- |
| SaV typing 1^st^ round | 30 min 50°C, 15 min 95°C [20 sec 94°C, 20 sec 42°C, 1 min 72°C]x20, 5 min 72°C. | SaV53a, SaV53b, SaV58 |
| SaV typing 2^nd^ round | 15 min 95°C [30 sec 94°C, 30 sec 42°C, 1 min 72°C]x30; 5 min 72°C | SaV55a, SaV55b, SaV58 |
| AiV typing 1^st^ round | 30 min 50°C, 15 min 95°C [30 sec 94°C, 30 sec 54°C, 1 min 72°C]x30, 5 min 72°C | AI68, AI70 |
| AiV typing 2^nd^ round | 15 min 95°C [30 sec 94°C, 30 sec 54 C, 1 min 72°C]x30, 5 min 72°C | AI69, AI71 |
