# Supplementary material for: Genetic Diversity of Enteric Viruses in Children under Five Years Old in Gabon
Source: Viruses. 2021 Mar 24;13(4):545. doi: 10.3390/v13040545 (PMC8064335; doi:10.3390/v13040545)
Supplement: Supplementary file 1 [file viruses-13-00545-s001.zip › viruses-111060_supplementary table1_revised.docx]

Supplementary table: primer and probes for detection and genotyping of different viral GE pathogens used in the study. Reference sequences are as follow^1^AB010145 (AiV-1), ^2^HQ398856 (HAstV1), ^3^FJ402983 (HAstV-MLB-1), ^4^X86557 (NoV GI), ^5^M87661 (NoV GII), ^6^M08661 (NoV GI), ^7^U07611 (NoV GII), ^8^JX459908 (NoV GII), ^9^AJ249939 (SaV GII), ^10^AF294739 (SaV GI), ^11^AY646856 (SaV GV.)

| **region** | **name** | **Sequence (5‘-3‘)** | **position** | **use** |
| --- | --- | --- | --- | --- |
| AiV (3CD) | AI1 (as) | AGGATGGGGTGGATRGGGGCAGAG | 6573-6550 ^1^ | Nested RT-PCR screening AiV |
|  | AI2 (s) | ACACTCCCACCTCCCGCCAGTA | 6261-6282 ^1^ |  |
|  | AI3 (as) | CCTTCGAAGGTCGCGGCRCGGTA | 6459-6437 ^1^ |  |
|  | AI4 (s) | GTACAAGGACATGCGGCG | 6280-6297 ^1^ |  |
| AiV (3CD) | AI68 (s) | GGAGTTCGGTGAAATCACCATTG | 6235-6257 ^1^ | Nested RT-PCR typing AiV |
|  | AI69 (s) | ACCTCCCGCCAGTACAAGGA | 6269-6288 ^1^ |  |
|  | AI70 (as) | CCAGGGCTCGGTGACGTC GCCT | 6814-6793 ^1^ |  |
|  | AI71 (as) | GGAAGAGCTGGGTGTCAAGATC | 6779-6758 ^1^ |  |
| AstV (ORF1b) | AV89a (s) | CATGGGAAGCTCCTRTGCT | 4192-4210 ^2^ | Semi-nested RT-PCR screening AstV |
|  | AV89b (s) | CATGGGAAACTTTTGTGCT | 3665-3683 ^3^ |  |
|  | AV89c (s) | TATGGGAAGCTCCTTTGCT | 4192-4210 ^2^ |  |
|  | AV90a (s) | TTTGGYATGTGGGTIAARCC | 3497-3516 ^3^ |  |
|  | AV90b (s) | TTTGGRATGTGGGTIAARCC | 3497-3516 ^3^ |  |
|  | AV90c (s) | TTTGGRATGTGGGTCAAGAG | 3497-3516 ^3^ |  |
|  | AV91 (as) | TTTGGWCCICCCCTCCA | 3834-3818 ^3^ |  |
| AstV (ORF1b) | AV91 (as) | TTTGGWCCICCCCTCCA | 4364-4348 ^2^ | Semi-nested RT-PCR typing AstV |
|  | AV92a (s) | GGTCARTGYGGGTGGTCACC | 3538-3557 ^2^ |  |
|  | AV93 (s) | GAYTGGACICGMTWTGATGG | 3628-3647 ^2^ |  |
| NoV (ORF1/2 junction) | NV192 (s) | GCYATGTTCCGCTGGATGC | 5282-5300 ^4^ | RT-qPCR screening NoV |
|  | NV192a (s) | GCAATGTTYCGCTGGATGC | 5282-5300 ^4^ |  |
|  | NV193 (as) | CGTCCTTAGACGCCATCATCA | 5379-5359 ^4^ |  |
|  | NV107e (s) | AACCAATGTTYAGMTGGATGAG | 5007-5028 ^5^ |  |
|  | NV107f (s) | AACCCATGTTCAGATGGATGAG | 5007-5028 ^5^ |  |
|  | NV107g (s) | AGGCCATGTTYAGRTGGATGAG | 5007-5028 ^5^ |  |
|  | NV107h (s) | AGCCAATGTTCAGATGGATGAG | 5007-5028 ^5^ |  |
|  | NV359 (as) | TCGACGCCATCTTCATTCACA | 5100-5080 ^5^ |  |
|  | NV-TM9 (as) | VIC-TGGACAGGAGATCGC-MGB-NFQ | 5321-5335 ^4^ |  |
|  | NV-TM15 (as) | FAM-TCGATCGCCCTCCCA-MGB-NFQ | 5062-5048 ^5^ |  |
| NoV (ORF1) | NV1c (s) | ATGAACATGAATGAGGATGG | 4499-4518 ^5^ | nested RT-PCR typing NoV |
|  | NV1d (s) | ATGAATATGAATGARGATGG | 4499-4518 ^5^ |  |
|  | NV1e (s) | ATGAATTCAATTGAGGATGG | 4499-4518 ^5^ |  |
|  | NV1f (s) | ATGAATGCAATTGAAGATGG | 4499-4518 ^5^ |  |
|  | NV7b (as) | GGDCCHTCASTYTTATC | 4977-4961 ^5^ |  |
|  | NV7c (as) | GGRCCYTCRCTYTTGTC | 4977-4961 ^5^ |  |
|  | NV7d (as) | GGTCCTTCTGATTTGTC | 4977-4961 ^5^ |  |
|  | NV7e (as) | GGCCCCTCRGTTTTGTC | 4977-4961 ^5^ |  |
|  | NV7f (as) | GGYCCTTCAGTYTTGTC | 4977-4961 ^5^ |  |
|  | NV6e (s) | ACCAYTWTGATGCAGACTA | 4554-4572 ^5^ |  |
|  | NV6f (s) | ACCAYTATGATGCTGATTA | 4554-4572 ^5^ |  |
|  | NV6g (s) | ATCAYTATGATGCWGAYTA | 4554-4572 ^5^ |  |
|  | NV4d (as) | ACYATCTCATCATCACCA | 4884-4866 ^5^ |  |
|  | NV4e (as) | ACGATCTCGTCRTCACCG | 4884-4866 ^5^ |  |
|  | NV4f (as) | ACTATYTCATCATCACCA | 4884-4866 ^5^ |  |
|  | NV4g (as) | ACGATCTCATCGTCCCCA | 4884-4866 ^5^ |  |
| NoV GI (ORF2 regionC) | NV360 (s) | CYATGTTCCGCTGGATGC G | 5283-5301 ^6^ | semi-nested RT-PCR typing NoV |
|  | NV360a (s) | CCATGTTCCGKTGGATGCG | 5283-5301 ^6^ |  |
|  | NV285a (as) | CCAACCCAYCCATTRTACAT | 5652-5671 ^6^ |  |
|  | NV285b (as) | CCGACCCAACCATTRTACAT | 5652-5671 ^6^ |  |
|  | NV362 (as) | TGRGGTGCCTGGACAAAGTT | 5532-5551 ^6^ |  |
|  | NV362a (as) | TGSGGTGMCTGGACAAAATT | 5532-5551 ^6^ |  |
|  | NV362b (as) | TGAGGGGCTTGAACATAATT | 5532-5551 ^6^ |  |
|  | NV362c (as) | TGGGGTGCCTGGACGAAGTT | 5532-5551 ^6^ |  |
|  | NV362d (as) | TCAGGGGCTTGGACAAAATT | 5532-5551 ^6^ |  |
| NoV GII (ORF2 regionC) | NV107e (s) | AACCAATGTTYAGMTGGATGAG | 5008-2028 ^7^ | semi-nested RT-PCR typing NoV |
|  | NV107f (s) | AACCCATGTTCAGATGGATGAG | 5008-2028 ^7^ |  |
|  | NV107g (s) | AGGCCATGTTYAGRTGGATGAG | 5008-2028 ^7^ |  |
|  | NV107h (s) | AGCCAATGTTCAGATGGATGAG | 5008-2028 ^7^ |  |
|  | NV107i (s) | AGTCAATGTTCAGATGGATGAG | 5008-2028 ^7^ |  |
|  | NV156a (as) | CCRGCATACCCATTRTACAT | 5386-5367 ^7^ |  |
|  | NV156b (as) | CCGCGTGCCCATTGTACAT | 5386-5367 ^7^ |  |
|  | NV156c (as) | CCKGCATAACCATTRTACAT | 5386-5367 ^7^ |  |
|  | NV364 (as) | TCTAATCCAAGGGTCTAT | 5243-5226 ^7^ |  |
|  | NV364a (as) | TCTWATCCAGGGRTCAAT | 5243-5226 ^7^ |  |
|  | NV364b (as) | CATAATCCAGGGATCAAT | 5243-5226 ^7^ |  |
| NoV GI (ORF2 P2-region) | NV351a (s) | CCICATGTIATTGCTGATGT | 5793-5812 ^5^ | semi-nested RT-PCR typing NoV |
|  | NV351b (s) | CCICACGTIATMGCAGATGT | 5793-5812 ^5^ |  |
|  | NV352a (as) | TTCCCACAGGCTTIAAYTG | 5793-5812 ^5^ |  |
|  | NV352b (as) | TTCCCACAGGCTTIAGYTG | 5793-5812 ^5^ |  |
|  | NV354 (s) | ATGATGATGGCGTCTAAGGAC | 5793-5812 ^5^ |  |
| NoV GII (ORF2 P2-region) | NV347a (s) | GAIGATGTCTTCACAGTYTCTT | 5661-5682 ^8^ | RT-PCR typing NoV |
|  | NV347b (s) | GATGATGTKTTCACWGTITCTT | 5661-5682 ^8^ |  |
|  | NV347c (s) | GATGAYGTITTCACIGTITCMT | 5661-5682 ^8^ |  |
|  | NV348a (as) | GGTTRACCCARGAATCAAA | 6651-6633 ^8^ |  |
|  | NV348b (as) | GRTTMACCCAAGAITCAAA | 6651-6633 ^8^ |  |
|  | NV348c (as) | GRTTRACCCAIACTTCAAA | 6651-6633 ^8^ |  |
| SaV (ORF1) | SaV56a (s) | GAYCAGGCTCTCGCCACCTA | 5078-5097 ^9^ | RT-qPCR screening SaV |
|  | SaV56b (s) | GATTTGGCCCTCGCCACCTA | 763-780 ^10^ |  |
|  | SaV57 (s) | TTTGAACAAGCTGTGGCATGCTAC | 5112-5135 ^11^ |  |
|  | SaV58 (as) | CCCTCCATYTCAAACACTA | 5183-5163 ^9^ |  |
|  | SaV-TM1 (as) | VIC-ACCACCTATRAACCA-MGB-BHQ-1 | 5119-5105 ^9^ |  |
|  | SaV-TM2 (as) | VIC-TGCCAC CAA TGT ACC A-MGB-BHQ-1 | 5157-5142 ^11^ |  |
| SaV (ORF1) | SaV53a (s) | TAGACTACAGCAAGTGGGA | 4356-4374 ^9^ | semi-nested RT-PCR typing SaV |
|  | SaV53b (s) | TGGAYTACTCYAAATGGGA | 38-56 ^10^ |  |
|  | SaV55a (s) | CCMTCKGGCATGCCATTCAC | 4529-4548 ^9^ |  |
|  | SaV55b (s) | CCATCAGGAATGCCCTTCAC | 211-230 ^10^ |  |
|  | SaV58 (as) | CCCTCCATYTCAAACACTA | 5183-5163 ^9^ |  |
